# Evaluation of venous thromboembolism prophylaxis protocol in hematopoietic cell transplant patients

**DOI:** 10.1038/s41409-023-02039-8

**Published:** 2023-08-25

**Authors:** Angela Lee, Corinne Badgley, Mimi Lo, Marisela Tan Banez, Larissa Graff, Lloyd Damon, Thomas Martin, John Dzundza, Melisa Wong, Rebecca Olin

**Affiliations:** 1 Department of Clinical Pharmacy, University of California, San Francisco, San Francisco, CA USA; 2 Division of Hematology-Oncology, University of California, San Francisco, San Francisco, CA USA; 3 Division of Hospital Medicine, University of California, San Francisco, San Francisco, CA USA

**Keywords:** Adverse effects, Risk factors

## Abstract

Hematopoietic cell transplant (HCT) recipients are at risk for thromboembolic and bleeding complications. There is limited evidence regarding the optimal approach to managing venous thromboembolism (VTE) prophylaxis in hospitalized patients undergoing HCT. In this retrospective cohort study, we evaluated the incidence of bleeding and VTE events in hospitalized HCT patients who received VTE prophylaxis per our institution’s VTE Prophylaxis Protocol (VPP), with either enoxaparin 40 mg subcutaneously daily or heparin 5 000 units subcutaneously twice daily, compared to historical controls who did not receive VTE prophylaxis. The primary outcome was a composite of major bleeding events, clinically relevant non-major bleeding (CRNMB), and minor bleeding. The secondary outcome was a composite of VTE events. A total of 614 patients were evaluated, including 278 prior to and 336 after implementation of VPP. VTE prophylaxis resulted in no difference in bleeding events (15.1% in the pre-VPP group vs. 14.6% in the post-VPP group, *p* = 0.86) or composite of major and CRNMB events (0.72% vs. 0.30%, *p* = 0.59). There was a trend toward lower incidence of VTE events in the post-VPP group which did not reach statistical significance (8.6% vs. 6.0%, *p* = 0.20). We conclude that VTE prophylaxis does not pose additional bleeding risk in HCT patients.

## Introduction

Hematopoietic cell transplant recipients are at high risk for thromboembolic complications before, during and after transplantation, yet are also at a high risk of bleeding due to prolonged chemotherapy-induced thrombocytopenia following conditioning chemotherapy. Therefore, anticoagulation management is challenging, as the risk of thrombosis must be carefully weighed relative to the risk of thromboprophylaxis. The overall incidence of VTE after HCT is 4–7%, while clinically symptomatic bleeding ranges between 15.2 and 27.1% [1–4]. Risk factors for VTE include hypercoagulability associated with malignancy, prolonged hospitalization, exposure to certain chemotherapies (especially high-dose myeloablative chemotherapy), the use of immunomodulatory drugs, infections, and graft-versus-host disease (GvHD) [1, 5, 6]. During hospitalization, the risk of VTE is commonly associated with the use of central venous catheters (CVC) such as peripherally-inserted central catheters (PICC), which may cause venous stasis and vessel injury [1, 7, 8]. The incidence of upper-extremity PICC-associated VTE is up to 7.8%, occurring in a median of 26 days after PICC placement [7–9].

Major guidelines recommend low molecular weight heparin (LMWH) or subcutaneous unfractionated heparin (UFH) for inpatient thromboprophylaxis in all hospitalized patients with active cancer without contraindications to anticoagulation [10–16]. However, the trials that supported these recommendations had limited representation of HCT patients [17, 18]. There is an informal consensus that routine pharmacologic VTE prophylaxis should not be offered to patients undergoing HCT despite insufficient evidence to support this recommendation [12]. Despite the high risk of VTE, HCT patients have not historically received VTE prophylaxis, due to the concern of thrombocytopenia and increased risk of bleeding [1–4]. Some studies have demonstrated that VTE prophylaxis did not increase bleeding risk in HCT patients [19, 20]. Thus, given the additional thrombotic risks associated with HCT, further studies are warranted. A retrospective review at the University of California, San Francisco (UCSF) Medical Center, comprising of malignant hematology and HCT admissions from 2009 to 2013, revealed that 59% of hospital-acquired VTE (HA-VTE) cases occurred at a platelet count of >50 × 10^9^/L, indicating that these patients could have been candidates for VTE prophylaxis [21]. This resulted in the development of our institution’s VTE Prophylaxis Protocol (VPP), in which patients eligible for VTE prophylaxis based on clinical parameters including platelet count >50 × 10^9^/L would receive standard prophylactic doses of enoxaparin or UFH [21].

As a part of routine quality improvement work, we sought to evaluate the safety and efficacy of the VPP in hospitalized HCT patients, by comparing rates of bleeding and HA-VTE events before and after the protocol’s implementation. We hypothesized that the VPP would result in no change to the incidence of bleeding, while decreasing the incidence of VTE.

## Materials/subjects and methods

### Study population

Patients eligible for inclusion in this study were adults 18 years or older who were admitted to UCSF for either an autologous or allogeneic HCT during the period of July 1, 2013, through July 1, 2017. The VPP was implemented on July 1, 2015, as part of a quality improvement project on the Malignant Hematology and Bone Marrow Transplant service. We retrospectively evaluated the pre-VPP group in the 24-month period prior to the implementation date in the HCT population, and the post-VPP group in a 24-month period following this date. We excluded patients who had contraindications to VTE prophylaxis as defined by the VPP, those receiving UFH 4 units/kilogram/h for veno-occlusive disease (VOD) prophylaxis, those receiving therapeutic anticoagulation at the time of admission, and those receiving outpatient HCT. The UCSF Institutional Review Board provided approval for this retrospective study. All study data were collected and managed using Research Electronic Data Capture (REDCap) tools.

### VTE prophylaxis protocol (VPP)

**Fig. 1:**
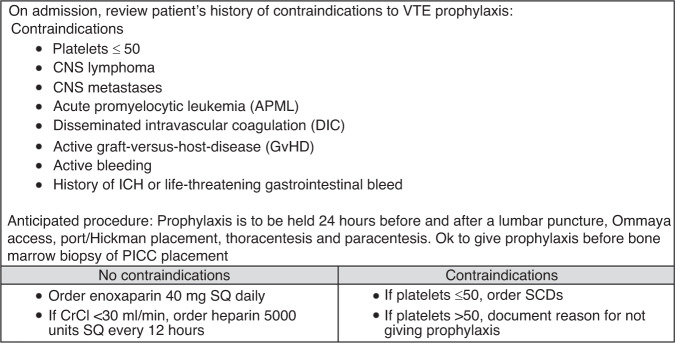
VTE prophylaxis protocol (VPP). Specifications of VTE prophylaxis protocol utilized at UCSF Medical Center.

All patients admitted to the Malignant Hematology and Bone Marrow Transplant Service were considered for standard doses of enoxaparin 40 mg subcutaneously (SQ) daily (if creatinine clearance was >30 ml/min) or UFH 5000 units SQ every 12 h (if creatinine clearance was ≤30 ml/min) (Fig. 1) [21]. Contraindications to pharmacologic VTE prophylaxis included the following: platelets ≤50 × 10^9^/L on admission, central nervous system (CNS) lymphoma or metastases, acute promyelocytic leukemia, disseminated intravascular coagulation, active GvHD, active bleeding, history of life-threatening gastrointestinal bleed or intracranial hemorrhage, or anticipated procedure [21]. As an institutional practice, prophylactic anticoagulation was not permitted in patients with CNS lymphoma or metastases due to guidelines recommending against its use, and limited data in HCT patients [10, 12, 16, 22]. Prophylaxis was to be held 24 h before and after a lumbar puncture, Ommaya access, port/Hickman placement, thoracentesis and paracentesis [21]. VTE prophylaxis was held during admission when platelets were ≤50 × 10^9^/L.

### Study design and endpoints

This was a single-center retrospective cohort study. Patients were identified by UCSF HCT data coordinators and a retrospective chart review was conducted. For patients that were admitted for more than one transplant, data for each admission were collected as an independent event.

The primary endpoint was the incidence of composite bleeding events, comprising of major bleeding, clinically relevant non-major bleed (CRNMB), and minor bleeding events, as defined by the International Society of Thrombosis and Hemostasis Criteria (ISTH) [23]. The ISTH definition for major bleeding comprises of fatal bleeding, and/or clinically symptomatic bleeding in a critical area or organ, and/or bleeding causing a fall in hemoglobin level of 2 g/dL (1.24 mmol/L) or more, and/or leading to transfusion of two or more units of whole blood or red blood cells. CRNMB events included any sign of hemorrhage that did not fit the criteria for major bleeding but met criteria for care escalation [23]. Minor bleeding events were any bleeding events outside our definition of major bleeding [23]. To confirm diagnosis of a bleeding event, provider documentation was referenced through notes, and reviewed by two reviewers to ensure accuracy.

The secondary endpoint was the incidence of HA-VTE, with a sub-analysis of CVC-associated VTE. Diagnosis of VTE events was confirmed by imaging confirmation through either Doppler ultrasound or computed tomography scan.

### Statistical analysis

Efficacy and safety outcomes between groups were analyzed using Pearson’s Chi-squared test or Fisher’s exact test. Analysis of risk factors associated with bleeding and VTE events was performed using univariate logistic regression. Our study was powered to detect a difference in incidence of composite bleeding events of 10%, using an alpha of 0.05 and a beta of 0.2 for at least 200 patients per group. Data was analyzed using STATA^©^ (StataCorp. 2017, release 15.1 College Station, TX, USA: StataCorp LP).

## Results

### Patient characteristics and VTE prophylaxis protocol

**Fig. 2:**
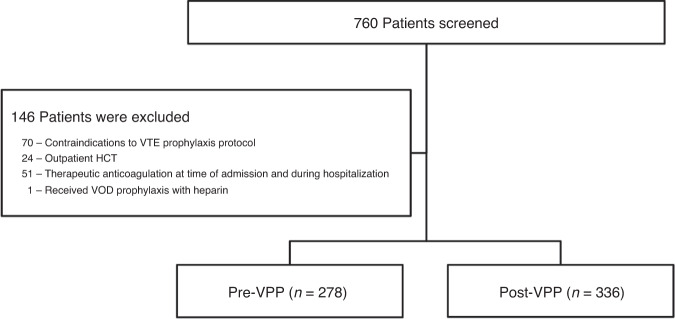
Patient CONSORT diagram. Flow diagram for patients included in analysis.

We identified 760 patients who received an allogeneic or autologous HCT during the 24 months prior to and 24 months after implementation of the VPP. Of these 760 patients, 146 were excluded for the following reasons: had contraindications per our VTE Prophylaxis Protocol (70), received therapeutic anticoagulation at the time of admission and during hospitalization (51), received outpatient HCT (24), or received VOD prophylaxis (1) (Fig. 2). VOD prophylaxis was not standardized at our institution during the study period. Only one patient, deemed to be high-risk of VOD per consensus guidelines, received VOD prophylaxis, and was excluded [24–26]. The most common contraindication to the VPP was platelets ≤50 × 10^9^/L at the time of admission. Thus, 614 patients who were eligible for VTE prophylaxis on admission were included in our analysis, of whom 278 were admitted pre-VPP and 336 were post-VPP. Of note, seven patients did receive VTE prophylaxis in the pre-VPP group, and 18 eligible patients did not receive VTE prophylaxis in the post-VPP group.

Baseline characteristics did not differ between groups (Table 1). In the post-VPP group, of the 318 patients that received VTE prophylaxis, 291 patients (91.5%) received enoxaparin and 27 patients (8.5%) received UFH. The median duration of VTE prophylaxis was 8 days in the post-VPP group (range 1–16). Median platelets at admission in the pre-VPP group were 180 × 10^9^/L (range 50–765) compared to 189 × 10^9^/L (range 52–759) in the post-VPP group (*p* = 0.02). No deaths were attributable to bleeding or VTE events in either group.

**Table 1:** Baseline characteristics.

Characteristic—no. (%)	Pre-VPP	Post-VPP	*P* value
	(*n* = 278)	(*n* = 336)	
Type of Transplant			0.27
Autologous HCT	194 (69.8)	248 (73.8)	
Allogeneic HCT	84 (30.2)	88 (26.2)	
Diagnosis			0.07
Acute Lymphoblastic Leukemia	16 (5.8)	20 (6.0)	
Acute Myeloid Leukemia	42 (15.1)	33 (9.8)	
Non-Hodgkin Lymphoma	45 (16.2)	36 (10.7)	
Multiple Myeloma, Amyloidosis	130 (46.8)	183 (54.5)	
Myelodysplastic syndromes	12 (4.3)	12 (3.6)	
Other^a^	33 (11.9)	52 (15.5)	
Conditioning Regimens			0.25
Myeloablative conditioning regimens	54 (19.4)	64 (19.0)	
Reduced intensity conditioning regimens	30 (10.8)	24 (7.1)	
Gender			
Male	164 (59.0)	208 (61.9)	0.46
Median Age at Time of Transplant (Years) [Min, Max]	58 [20,76]	55 [19,77]	0.26
BMI			
≥30 kg/m^2^	78 (28.1)	103 (30.7)	0.48
GFR Range			
≥30 ml/min	269 (96.8)	321 (95.5)	0.72
KPS Score			0.06
90–100	229 (82.4)	299 (89.0)	
50–80	48 (17.3)	37 (11.0)	
Median Duration of Hospitalization (Days) [Min, Max]	20 [7, 107]	18 [11,78]	<0.001
Prior VTE (within 1 year prior to transplant date)	19 (6.8)	25 (7.4)	0.77
Current Smoker or Former Heavy Smoker (>20 pack-years)	20 (7.2)	29 (8.6)	0.51
Medications Causing Clotting Risk (within 6 months of transplant)—Any^b^	86 (30.9)	84 (25.0)	0.10
Medications Causing Clotting Risk (within 6 months of transplant)—By medication^b^			0.70
Estrogens	7/86 (8.1)	4/84 (4.8)	
Progestins	3/86 (3.5)	1/84 (1.2)	
Erythropoietin	3/86 (3.5)	2/84 (2.4)	
Lenalidomide	65/86 (75.6)	69/84 (82.1)	
Pomalidomide	4/86 (4.7)	7/84 (8.3)	
Sirolimus^c^	3/86 (3.5)	2/84 (2.4)	
Pegaspargase	1/86 (1.2)	0/84 (0.0)	
Central Line Type (Placed on Admission) - Total	220 (79.1)	288 (85.7)	0.03
Central Line Type (Placed on Admission) - By line type			0.38
PICC	157/220 (71.4)	219/288 (76.0)	
Hickman	51/220 (23.2)	59/288 (20.5)	
Port/Port-a-cath	12/220 (5.5)	10/288 (3.5)	
Multiple Central Lines	44 (15.8)	52 (15.5)	0.91
Central Line Type (Regardless of Time of Placement)			0.54
PICC	175 (62.9)	223 (66.4)	
Hickman	61 (21.9)	72 (21.4)	
Port/Port-a-cath	42 (15.1)	41 (12.2)	

^a^Other Diagnoses include: chronic myeloid leukemia, chronic lymphoblastic leukemia, biphenotypic or mixed phenotype acute leukemia, germ cell tumor, Hodgkin’s lymphoma, non-malignant hematologic disorders, medulloblastoma, aplastic anemia.

^b^Medications causing clotting risk: one patient in the post-VPP group received ≥1 medications (lenalidomide and pomalidomide) causing clotting risk (within 6 months of transplant).

^c^Sirolimus in combination with tacrolimus for GvHD prophylaxis.

### Bleeding events

The primary outcome of composite bleeding events (major bleeding, CRNMB, and minor bleeding) occurred in 42 of 278 patients (15.1%) in the pre-VPP group, and in 49 of 336 patients (14.6%) in the post-VPP group (*p* = 0.86) (Table 2). Among the bleeding events observed, major bleeding and CRNMB were uncommon and did not differ between the pre-VPP group and post-VPP group (2 of 278 (0.72%) vs. 1 of 336 (0.30%), *p* = 0.59; Table 2). In the pre-VPP group, there was one major bleed (retroperitoneal bleed), and one clinically relevant non-major bleed (upper gastrointestinal (GI) bleed). In the post-VPP group, there was one major upper GI bleed. No fatal bleeding events occurred in either group.

**Table 2:** Bleeding events.

Outcome —no. (%)	Pre-VPP	Post-VPP	*P* value
	(*n* = 278)	(*n* = 336)	
Composite Bleeding Event	42 (15.1)	49 (14.6)	0.86
Major Bleeding and Clinically Relevant Non-Major Bleeding Event	2 (0.72)	1 (0.30)	0.59
Minor Bleeding Event	40 (14.4)	48 (14.3)	1.00
Intervention Given for Bleeding Event	9/42 (21.4)	11/49 (22.4)	0.56
Platelet Transfusions Given for Bleeding Event	11/42 (26.2)	18/49 (36.7)	0.28

The median platelet level at time of bleeding event was 31 × 10^9^/L (range 6-319) in the pre-VPP group, and 32 × 10^9^/L (range 3–752) in the post-VPP group (*p* = 0.87). No patients were on concomitant medications that increased bleeding risk, such as aspirin, non-steroidal anti-inflammatory drugs, or P2Y12 inhibitors. Of the 49 bleeding events in the post-VPP group, 6 patients still had active orders for VTE prophylaxis at the time of the bleeding event, which were subsequently discontinued after the bleeding event was documented. Of these six patients, five had platelets >50 × 10^9^/L at the time of bleeding event (which was an appropriate threshold to continue prophylaxis per the VPP), and one of six patients had platelets <50 × 10^9^/L at the time of bleeding event (which was not compliant with the VPP).

### Thrombotic events

In the pre-VPP group, 24 of 278 (8.6%) patients experienced VTE events, compared to 20 of 336 (6.0%) patients in the post-VPP group (*p* = 0.20; Table 3). In both groups, the majority of VTE events were line-associated VTEs (Table 3). No incidence of VTE events were manifestations of VOD or transplant-associated thrombotic microangiopathy. In the pre-VPP group, the eight non-line associated VTE events included three pulmonary embolisms, two superficial clots, two lower extremity DVTs, and one right atrial thrombus. In the post-VPP group, the five non-line associated VTE events included three pulmonary embolisms, and one upper extremity DVT, and one lower extremity DVT. The majority of VTE events occurred while patients were off VTE prophylaxis (17 of 20 VTE events in the post-VPP group). Line-associated VTEs occurred while off VTE prophylaxis in 14 of 16 post-VPP patients. Non-line associated VTEs occurred while off VTE prophylaxis in four of five post-VPP patients.

**Table 3:** Thrombotic events.

Outcome —no. (%)	Pre-VPP	Post-VPP	*P* value
	(*n* = 278)	(*n* = 336)	
VTE Incidence	24 (8.6)	20 (6.0)	0.20
Median Time of VTE Diagnosis from Admission - Days [Min, Max]	11.5 [2,70]	13 [2,24]	0.80
Line-Associated VTE	16 (5.8)	15 (4.5)	0.58
Non-Line Associated VTE	8 (2.9)	5 (1.5)	0.27
Outcome —no. (%)	Pre-VPP patients with VTE event (*n* = 24)	Post-VPP patients with VTE event (*n* = 20)	*P* value
Type of VTE Event			0.39
Upper Extremity DVT	15/24 (62.5)	16/20 (80.0)	
Lower extremity DVT	3/24 (12.5)	1/20 (5.0)	
Pulmonary Embolism	3/24 (12.5)	3/20 (15.0)	
Other	3/24 (12.5)^a^	0/20 (0.0)	

^a^Pre-VPP (Other): includes superficial clot (2), right atrial thrombus (1).

The median platelet level at the time of VTE diagnosis was 59 × 10^9^/L (range 14-206) in the pre-VPP group, and 60 × 10^9^/L (range 24–167) in the post-VPP group (*p* = 0.63). Of the 24 patients that experienced a VTE event in the pre-VPP group, 2 patients (8.3%) had a prior cancer-associated VTE event, compared to 1 of 20 patients (5.0%) in the post-VPP group (*p* = 0.78). The time between admission and VTE diagnosis was a median of 11.5 days (range 2–70) in the pre-VPP group, compared to 13 days (range 2–24) in the post-VPP group (*p* = 0.80). For the patients who experienced a VTE, the Khorana Risk Score was calculated [27]. Most patients were found to have Khorana Risk Score < 3 (low to intermediate risk), and no difference was found between pre- and post-VPP groups (*p* = 0.22).

### Sensitivity analysis

A sensitivity analysis was performed, which excluded patients who received pharmacologic prophylaxis prior to the initiation of the protocol implementation date (*n* = 7), and any eligible patients that were not given prophylaxis following the protocol’s implementation (*n* = 18). After excluding these 25 patients, 589 patients were included in the sensitivity analysis. There was no difference in composite bleeding events between groups (pre-VPP: 40 of 271 (14.8%) vs. post-VPP: 49 of 318 (15.4%), *p* = 0.83), or in VTE events (pre-VPP: 22 of 271 (8.1%) vs. post-VPP: 19 of 318 (6.0%), *p* = 0.31).

### Subgroup analysis of primary and secondary outcomes

In patients that experienced a bleeding or VTE event, univariate logistic regression analyses were utilized to evaluate risk factors that may be associated with each outcome in the post-VPP group. Duration of VTE prophylaxis (measured in days) was not associated with an increased risk of bleeding (OR 1.06; 95% CI, 0.95–1.18, *p* = 0.28), but was associated with decreased incidence of VTE events (OR 0.74, 95% CI, 0.62 to 0.89, *p* = 0.001) (Table 4). PICC lines were associated with an increased risk of VTE events (OR 5.15, 95% CI, 1.17–22.6, *p* = 0.008), while multiple lines were not associated with an increased risk of VTE events (OR 0.96, 95% CI, 0.27–3.41, *p* = 0.95) (Table 4). Patients with an allogeneic HCT demonstrated an increased risk of bleeding (OR 3.35, 95% CI, 1.79–6.25, *p* = 0.0002), and decreased risk of VTE events (OR 0.69, 95% CI, 0.22–2.12, *p* = 0.50), compared to those with an autologous HCT (Table 4). Most patients in our study received an autologous HCT. However, because patients with an allogeneic HCT are subject to more complications such as GvHD, and prolonged thrombocytopenia and hospitalization, that may predispose them to greater risk of bleeding or VTE, we performed a subgroup analysis in this population. Similar to the results of the overall cohort, the allogeneic HCT subgroup demonstrated no difference in composite bleeding events or VTE events between the pre-VPP and post-VPP groups, though there was a numerical trend towards more bleeding events (18 (21.4%) vs. 24 (27.3%), *p* = 0.37) and less VTE events in the post-VPP group (8 (9.5%) vs. 4 (4.5%), *p* = 0.24) (Tables S1, S2).

**Table 4:** Post-VPP Group: clinical characteristics associated with bleeding events or VTE events in univariate logistic analysis.

	Post-VPP Group: Patients with Bleeding Events (*n* = 49)		Post-VPP Group: Patients with VTE Events (*n* = 20)	
	vs. Patients with no Bleeding Events (*n* = 287)		vs. Patients with no VTE Events (*n* = 316)	
Variable	OR (95% CI)	*P* value	OR (95% CI)	*P* value
Duration of VTE prophylaxis (Per day)	1.06 (0.95–1.18)	0.28	0.74 (0.62–0.89)	0.001
Age ≥65 years old	0.89 (0.68–1.17)	0.31	1.00 (0.86–1.17)	0.99
BMI ≥ 30	1.06 (0.97–1.16)	0.23	1.00 (0.87–1.15)	0.95
HCT Type (Allogeneic vs. Autologous)	3.35 (1.79–6.25)	0.0002	0.69 (0.22–2.12)	0.50
Line type at admission: PICC	N/A	N/A	5.15 (1.17–22.6)	0.008
Multiple Lines	N/A	N/A	0.96 (0.27–3.41)	0.95

## Discussion

This study represents the one of the largest analyses of the safety and efficacy of VTE prophylaxis in the hospitalized HCT population to date. We found no significant difference in composite bleeding events between groups, suggesting that VTE prophylaxis appeared to pose no additional bleeding risk in this population, with a trend towards decreased VTE events.

Few studies have evaluated the incidence of bleeding events in HCT patients on concurrent thromboprophylaxis. Notably, the rates of bleeding in our cohort (15.1% pre-VPP and 14.6% post-VPP) were similar to those observed in other analyses, in which patients did not receive any thromboprophylaxis (14.3–27.1%) [1, 2, 28]. In contrast, Ibrahim et al. observed a 23% incidence of any bleeding event in a case series of 26 patients that received at least one dose of enoxaparin (mean dose: 0.5 mg/kg/day) with a mean platelet count of 55 × 10^9^/L [19]. This incidence of bleeding events is higher compared to our cohort and may be attributed to enoxaparin administration during thrombocytopenia. Our VPP protocol excluded patients that had platelets <50 × 10^9^/L, and held thromboprophylaxis when platelet dropped below <50 during admission, which could explain the lower bleeding rates and improved safety compared to Ibrahim et al. Other studies have evaluated VTE prophylaxis for VOD prevention in the HCT population, and demonstrated that LMWH did not appear to increase bleeding risk, with rates of bleeding comparable to that found in our study [29].

The administration of VTE prophylaxis resulted in a trend toward decreased VTE between the pre-VPP and post-VPP groups. Rates of VTE in our study were 6.0% in the post-VPP group, which is similar to the rates of 4–7% as reported in metanalyses [30, 31]. The similar rates of VTE in our study, despite the fact that patients received thromboprophylaxis, reinforces the findings of prior studies that suggests that LMWH does not decrease the incidence of catheter-associated thrombosis in cancer patients [27, 29]. Similar to prior reports, catheter-associated thrombosis events were more common than systemic VTE [32, 33]. However, it is notable that a longer duration of VTE prophylaxis was associated with a decreased likelihood of VTE risk in our study. The median time to VTE event observed in our study was shorter compared to prior reported outcomes in HCT patients (11.5–13 days vs. 12–35 days) [1, 13, 34]. The shorter time to VTE event may be attributed to the fact that 15.5–15.8% patients had multiple lines upon admission (i.e., patients had a line prior to admission, with an additional line inserted at admission), or had PICC lines (71.4–76.0%) at admission. Although our univariate analysis showed that PICC lines increased VTE risk between patients that experienced VTE events in the post-VPP group, the presence of multiple lines did not demonstrate this. Due to small sample size and low incidence of VTE events overall, it is premature to conclude that thromboprophylaxis provides no benefit in reducing VTE events; a larger cohort would be needed to definitively assess its benefit in this patient population.

Our study has several limitations. Retrospective chart review may have underestimated the frequency and exact timing of bleeding or clotting events. Despite this limitation, the rates of bleeding in our study did not differ substantially from existing literature. Diagnosis of VTE events was confirmed through imaging; however, initial diagnosis relied on symptomatic presentation, and corresponding provider evaluation. The low incidence of VTE events and modest sample size reduced the power of our study to detect a statistically significant difference in VTE events from the use of prophylaxis. Furthermore, no patients received VTE prophylaxis throughout the entire duration of their hospitalization, due to the expected profound thrombocytopenia following conditioning chemotherapy and the design of the VPP to discontinue VTE prophylaxis when platelets <50 × 10^9^/L. Additionally, patients were not restarted on thromboprophylaxis if platelets recovered to >50 × 10^9^/L. Therefore, it is difficult to assess the full efficacy of this intervention, as patients had variable exposure to anticoagulation and routine imaging was not performed in all patients. Lastly, the UFH thromboprophylaxis dosing of 5000 units SQ every 12 h  was a standardized protocol based on provider preference at our institution. Adjustment to 5000 units every 8 h for patients weighing greater than 50 kg was not considered [35]. Therefore, it is possible that some patients could have been relatively underdosed; the effects of this on bleeding and VTE rates cannot be determined.

In this single-institution retrospective study, pharmacologic VTE prophylaxis, under clinically appropriate circumstances, and within the specifications of our protocol, did not increase the risk of bleeding in HCT patients. As such, VTE prophylaxis has continued to be a standard of care practice at our institution. There was an observed trend toward decreased incidence of VTE, though larger studies utilizing serial imaging to assess VTE will be required to confirm this benefit.

### Supplementary information

Table S1 and Table S2

## Data Availability

Data sharing not applicable to this article as no datasets were generated or analyzed during the current study.
