# Supplementary material for: Evaluation of venous thromboembolism prophylaxis protocol in hematopoietic cell transplant patients
Source: Bone Marrow Transplant. 2023 Aug 25;58(11):1247–53. doi: 10.1038/s41409-023-02039-8 (PMC10622316; doi:10.1038/s41409-023-02039-8)
Supplement: Supplementary file 1 — Table S1 and Table S2 [file 41409_2023_2039_MOESM1_ESM.pdf]

Supplemental Materials: Bleeding and Thrombotic Event Outcomes in Allogeneic Hematopoietic Stem Cell Transplant (HCT) Subgroup

Table S1: Bleeding Events in Allogeneic HCT Patients

Outcome —no. (%)	Pre-VPP (n=84)	Post-VPP (n=88)	P-value
**Composite Bleeding Events**	18 (21.4)	24 (27.3)	0.37
**Major Bleeding and Clinically Relevant Non-Major Bleeding Event**	2 (2.4)	0 (0.0)	0.24
**Minor Bleeding Event**	16 (19.0)	24 (27.3)	0.20
**Intervention Given for Bleeding Event**	5/18 (27.8)	4/24 (16.7)	0.46
**Platelet Transfusions Given for Bleeding Event**	5/18 (27.8)	13/24 (54.2)	0.12

Table S2: Thrombotic Events in Allogeneic HCT Patients

Outcome —no. (%)	Pre-VPP (n=84)	Post-VPP (n=88)	P-value
**VTE Incidence**	8 (9.5)	4 (4.5)	0.24
**Median Time of VTE Diagnosis from Admission – Days [Min, Max]**	12 [2, 70]	9 [3, 24]	0.67
**Line-Associated VTE**	5 (6.0)	4 (4.5)	0.74
**Non-Line Associated VTE**	3 (3.6)	0 (0.0)	0.11
**Outcome —no. (%)**	**Pre-VPP patients with VTE event (n=8)**	**Post-VPP patients with VTE event (n=4)**	**P-value**
**Type of VTE Event**			0.77
Upper Extremity DVT	4/8 (50.0)	4/4 (100.0)	
Lower extremity DVT	1/8 (12.5)	0/4 (0.0)	
Pulmonary Embolism	2/8 (25.0)	0/4 (0.0)	
Other	1/8 (12.5)	0/4 (0.0)	
